# Prevalence and incidence of non-gout crystal arthropathy in southern Sweden

**DOI:** 10.1186/s13075-019-2077-6

**Published:** 2019-12-17

**Authors:** Mohaned Hameed, Aleksandra Turkiewicz, Martin Englund, Lennart Jacobsson, Meliha C. Kapetanovic

**Affiliations:** 1 Department of Clinical Sciences, Lund, Section for Rheumatology, Lund University, Lund and Skåne University Hospital, Lund, Sweden; 2 Department of Clinical Sciences, Lund, Section for Rheumatology, Lund University, Lund and Skåne University Hospital, Gothenburg, Sweden; 3 Clinical Epidemiology Unit, Orthopaedics, Department of Clinical Sciences Lund, Lund University, Lund, Sweden; 4 Clinical Epidemiology Research and Training Unit, Boston University School of Medicine, Boston, MA USA; 5 Department of Rheumatology & Inflammation Research, Institute of Medicine, The Sahlgrenska Academy, University of Gothenburg, Gothenburg, Sweden

**Keywords:** Non-gout crystal arthropathy, Calcium pyrophosphate crystal deposition disease (CPPD), Unspecified non-gout crystal deposition disease, Prevalence, Incidence

## Abstract

**Objective:**

To estimate the prevalence and incidence of non-gout crystal arthropathy in relation to socioeconomic factors in southern Sweden.

**Methods:**

All patients (age ≥ 18 years) with at least one visit to a physician with the diagnosis of interest in the Skåne region (population of 1.3 million) in 1998–2014 were identified. Non-gout crystal arthropathy (ICD-10 codes M11.0–M11.9) was subclassified in four different groups: calcium pyrophosphate crystal deposition related arthropathy (CPPD), unspecified non-gout arthropathies, chondrocalcinosis, and hydroxyapatite crystal deposition disease. The crude and age-adjusted point prevalence on December 31, 2014, and the cumulative incidence during 2014 were calculated for all non-gout crystal arthropathies, CPPD, and other unspecified non-gout arthropathies overall and in relation to occupation, income, and level of education.

**Results:**

The crude 2014 point prevalence (95% CI) and 2014 cumulative incidence (95% CI) of all non-gout crystal arthropathies were 0.23% (0.23–0.24) and 21.5 (19–25) cases/100,000 persons. Mean age (range) among all prevalent cases in 2014 was 71 (20–102) years and 56% were males. The point prevalence and cumulative incidence of CPPD were 0.09% (0.08–0.09) and 8 (7–10)/100,000 persons, respectively. The corresponding data for unspecified non-gout crystal deposition disease was 0.16% (0.16–0.17) and 15.6 (13–18)/100,000 persons, respectively. The prevalence and incidence of CPPD and unspecified non-gout crystal arthropathies were slightly higher in men and increased with age irrespective of gender. Unspecified non-gout crystal arthropathy but not CPPD was less prevalent in persons with ≥ 15 years of education, whereas there were no clear associations with occupation and income.

**Conclusion:**

The prevalence of all diagnosed non-gout crystal arthropathies was 0.23%, thus considerably less prevalent than gout in southern Sweden. CPPD and other unspecified non-gout crystal arthropathies are the predominant diagnoses, increasing with age and in men. With the exception for unspecified non-gout crystal arthropathies being inversely correlated to a higher level of education, no convincing association with the socioeconomic factors was found.

## Background

Crystal arthropathies excluding gout (non-gout crystal arthropathies) include acute and chronic calcium pyrophosphate, hydroxyapatite, and other related crystal deposition diseases. In the International Statistical Classification of Diseases Tenth Revision (ICD-10), these conditions are categorized as other unspecified crystal arthropathies (M11.0–M11.9 codes)*.* Calcium pyrophosphate crystal deposition related arthropathy (CPPD) is the most common non-gout crystal arthropathy and is by the EULAR proposed to be an umbrella term for all calcium pyrophosphate crystal (CPP) arthropathies [1]. CPPD can be present clinically as CPPD-associated osteoarthritis, acute CPP crystal arthritis, or chronic CPP crystal arthritis [1, 2]. The most common clinical form of CPPD is acute CPP crystal arthritis affecting knee joint, wrist, or shoulder in person older than 65 years (previously known as pseudogout). A higher age is strongly associated with CPPD [1–4] and CPPD is uncommon under age 60 years [1, 2]. An onset of CPPD before age 45 is usually associated with genetic (i.e., familial CPPD) or metabolic factors [1–9]. Chondrocalcinosis (CC), a usually asymptomatic CPPD commonly visualized on radiographs, supports the diagnosis but the identification of rhomboid, mostly intracellular calcium pyrophosphate crystals in the synovial fluid, is necessary for confirmation of diagnosis [1–4]. Epidemiological studies of radiographic signs of chondrocalcinosis in the knees have shown a prevalence of 7% to 10% in people approximately 60–70 years when either hand/wrist or hip/symphysis pubis were examined [3, 4, 10–12]. The incidence and prevalence of clinically diagnosed forms of CPPD disease are less well established.

Hydroxyapatite crystal deposition is less common than CPPD and may clinically present as calcific tendinitis, peritendinitis and periarthritis calcarea, or an appetite-associated destructive arthropathy (i.e., Milwaukee shoulder) [12–15]. It is caused by intra- and/or periarticular deposition of hydroxyapatite crystals most often affecting the shoulder joint but can also involve other peripheral joints or spine. It is most common in the middle-aged people but occasionally occurs in younger ages [12–15].

Both the prevalence and incidence and their association to socioeconomic factors are insufficiently studied. The ProVA study from northeastern Italy reported no relevant differences in income and level of education between elderly people with knee and hip chondrocalcinosis and controls [16].

The aims of the present study were (1) to examine the prevalence and the incidence of diagnosed non-gout crystal arthropathy and (2) to investigate the possible associations of non-gout crystal arthropathy with socioeconomic factors such as level of education, income, or age in adult residents in southern Sweden.

## Methods

### Data sources

The Skåne Healthcare Register (SHR) includes information on all health care visits with given ICD10-coded diagnoses for all citizens in the Skåne region (total population of 1.3 million). All adult (≥ 18 years) residents in the Skåne region in the year 2014, who between 1998 and 2014 had received at least one diagnosis of non-gout crystal arthropathy (ICD-10 codes M11.0–M11.9) by any physician, were identified.

Socioeconomic variables were retrieved from the Statistics Sweden [17] and data on age, sex, and residential area from the national Swedish population register. All information was linked on an individual level using the Swedish encrypted 10-digit personal identification number.

### Definition of non-gout crystal arthropathy

A person was considered to have non-gout crystal arthropathy if any M11 code according to the ICD-10 had been registered at least once by any physician (within primary, secondary or in-patient health care). The date of first such visit was considered as the date for diagnosis of non-gout crystal arthropathy. All diagnostic codes were stratified in three different groups: (1) CPPD (M11.8, M11.8B, M11.8C, M11.8D, M11.8F, M11.8G, M11.8H, and M11.8X) + chondrocalcinosis (M11.1 and M11.2); (2) hydroxyapatite crystal deposition disease (M11.0); and (3) unspecified other non-gout arthropathies (M11.9, M11.9B, M11.9C, M11.9D, M11.9F, M11.9G, M11.9H, M11.9X, and M11-).

Due to the limited number of persons with hydroxyapatite crystal deposition disease and chondrocalcinosis in the registry (*n* = 24), these arthropathies were excluded from the main analyses.

### Socioeconomic status

Occupation was categorized according to the Swedish standard system for classification of occupations (Standard för Svensk Yrkes Klassificering 1996; SSYK96) [18], which is based on International Standard Classification of Occupations (ISCO-88) [19].

The occupations were classified in the following four groups: high-skilled white-collar (managers, professionals and technicians, and associated professionals), low-skilled white-collar (clerical support workers and service and sales workers), high-skilled blue-collar (skilled agricultural, forestry and fishery workers, and craft and related trades workers), and low-skilled blue-collar (plant and machine operators and assemblers and occupations with demand of elementary education).

Level of education was classified in four groups according to years of education: 0–9 years (primary school), 10–12 years (high school), 13–14 years (upper secondary school), and ≥ 15 years (university degree).

Level of income was divided in three groups: lowest quartile (low income corresponding to < 11,270€/year), second and third quartile (middle income 11,270–27,690€/year), and highest quartile (high income > 27,270€/year).

### Missing data

Complete information on age and sex was available. Education and income data were missing in 1.3% of subjects, and occupation data were missing in 22.6% of cases (15.5% of persons aged ≥ 35 years). The average in Skåne Healthcare Register in years between 1998 and 2014 was 9.7% of visit in specialist care and about 38.5% of visits within primary care had the diagnostic code missing. These missing data have been decreased with time to about 0.58% and 6.71% at year 2014 for special care respectively primary care. No validation of diagnoses by review of clinical records was done.

#### Statistical methods

The 2014 point prevalence was defined as the proportion of all participants ≥ 18 years, who are still residents of the Skåne region on 31 December 2014, having fulfilled our non-gout crystal arthropathy definition between 1 January 1998 and 31 December 2014.

We calculated the cumulative incidence as the proportion of all participants ≥ 18 years, who were residents of the Skåne region by 31 December 2013, fulfilling our criterion for non-gout crystal arthropathy between 1 January 2014 and 31 December 2014 among those with no physician-diagnosed such diagnosis in the region between 1 January 1998 and 31 December 2013.

In addition, the distribution of the different M11 sub-codes was reported.

Prevalence and cumulative incidence (for the year 2014) was calculated stratified by age groups, sex, and socioeconomic status.

Sensitivity analysis was performed using a more stringent definition, i.e., at least two diagnoses from a physician or at least one from a specialist was also applied.

## Results

In total, there were 2393 persons with prevalent non-gout crystal arthropathy in southern Sweden and 217 persons with incident disease in 2014 in a population of 1,022,545. The crude 2014 point prevalence (95% CI) and 2014 cumulative incidence (95% CI) of all non-gout crystal arthropathies were 0.23% (0.23–0.24) and 21.5 [2, 19–24] cases per 100,000 persons. The mean age (range) for a person diagnosed with non-gout crystal arthropathy in the year 2014 was 71 years (20–102) and 56% were men.

The number of subjects in 2014 with prevalent incident disease and the relative distribution (percentage of all subjects with a diagnosis) of the major sub-diagnoses of non-gout crystal arthropathies are given in Table 1. The distributions of sub-diagnoses were overall similar for prevalent and incident cases in 2014 (Table 1). The absolute majority were diagnoses with either CPPD or “unspecified other non-gout arthropathies”, 96% and 97.5% in specialized non-primary care and primary care, respectively.

**Table 1:** Number of subjects diagnosed with non-gout crystal arthropathy (ICD10 M11) and relative distributions of subtypes for both prevalent cases (*N* = 2393) and incident cases (*N* = 2017) in 2014. Only the first (incident) diagnosis for 217 incident cases is listed. Diagnosis among (prevalent) cases-only the latest diagnosis included one diagnosis per person

	IC10 diagnostic codes	The numbers and distribution of subjects diagnosed with the various subcodes within M11 group among prevalent cases number (%)	The numbers and distribution of subjects diagnosed with the various subcodes within M11 group among incident cases number (%)
All non-gout crystal arthropathies		2393 (100%)	217 (100%)
Calcium pyrophosphate crystal deposition related arthropathy	M11.8 calcium pyrophosphate arthropathy	216 (9.03%)	26 (8.13%)
	M11.8B in shoulder joint	9 (0.38%)	2 (0.63%)
	M11.8C in elbow joint	14 (0.59%)	3 (0.94%)
	M11.8D in wrist joint/hand	61 (2.55%)	20 (6.25%)
	M11.8F in hip joint	11 (0.46%)	2 (0.63%)
	M11.8G in knee joint	352 (14.71%)	48 (15.0%)
	M11.8H in ankle and foot	31 (1.30%)	7 (2.19%)
	M11.8X site unspecified	12 (0.5%)	10 (3.13%)
Chondrocalcinosis	M11.1 familial chondrocalcinosis	7 (0.29%)	0
	M11.2 other chondrocalcinosis	68 (2.84%)	3 (0.94%)
Hydroxyapatite crystal deposition disease	M11.0 hydroxyapatite deposition disease	22 (0.92%)	5 (1.56%)
Unspecified other non-gout arthropathies	M11.9 crystal arthropathy, unspecified	289 (12.08%)	36 (11.25%)
	M11.9B crystal arthropathy, unspecified in shoulder joint	23 (0.96%)	2 (0.63%)
	M11.9C crystal arthropathy, unspecified in elbow joint	18 (0.75%)	5 (1.56%)
	M11.9D Crystal arthropathy, unspecified in wrist joint/ hand	120 (5.01%)	20 (6.25%)
	M11.9F crystal arthropathy, unspecified in hip joint	33 (1.38%)	2 (0.63%)
	M11.9G crystal arthropathy, unspecified in knee joint	434 (18.14%)	37 (11.56%)
	M11.9H crystal arthropathy, unspecified in ankle and foot	199 (8.32%)	12 (3.75%)
	M11.9X crystal arthropathy, unspecified with unspecified site	9 (0.38%)	5 (1.56%)
	M11- other crystal arthritis, unspecified	465 (19.43%)	75 (23.4%)

A separate analysis applying a more stringent definition, i.e., at least two diagnoses from a physician within primary care or at least one from a specialist, was also applied resulting in overall similar results (data not shown). In total, 86% of all individuals with non-gout crystal arthropathy diagnosis fulfilled this definition also.

Of the 2393 subjects with prevalent non-gout crystal arthropathy in 2014, 82% had at least once been diagnosed in specialized non-primary care. In specialized non-primary care, the most common diagnoses were M11.8 (“calcium pyrophosphate crystal deposition related arthropathy”) and M.11.9 (“unspecified other non-gout arthropathies”) used at 93% of the visits, whereas in primary care, the M11- (“other crystal arthritis, unspecified”) was used at 93% of the visits. In 7.2% of all prevalent cases, different diagnoses within the M11 group of diagnoses had been given at different visits. The most common combinations of diagnoses were “unspecified other non-gout arthropathies” (M11.9) and “calcium pyrophosphate crystal deposition related arthropathy” (M11.8) both registered in 3.6% and “other crystal arthritis, unspecified” (M11-) and “calcium pyrophosphate crystal deposition related arthropathy” (M11.8) both registered in 2.1% subjects.

The prevalence and cumulative incidence of patients diagnosed with CPPD in 2014 were 0.09% (95% CI 0.08–0.09) and 8 (95% CI 6.4–10)/100,000 persons, respectively. Men had generally both higher prevalence and incidence of CPPD in all age categories, compared to women (Table 2).

**Table 2:** Point prevalence and cumulative incidence of calcium pyrophosphate crystal arthropathy (CPPD) in Skåne, in 2014, age and sex stratified

		Prevalence				Cumulative incidence			
Sex	Age group	Number of persons with diagnosis	Population (*n*)	Prevalence/100,000 persons	95% CI	Number of persons with diagnosis	Population (*n*)	Cumulative incidence/100,000 persons	95% CI
All	≥ 18	869	1,022,545	85	80–91	81	1,012,218	8	6–10
Women	All ≥ 18	383	519,302	74	67–82	31	514,766	6	4–8
Women	18–34	3	143,725	209	40–645	0	143,160	0	0–3
Women	35–44	8	83,016	964	451–1939	0	82,342	0	0–6
Women	45–54	20	83,240	2403	1531–3736	1	82,315	2	0–8
Women	55–64	46	73,596	6250	4668–8354	4	73,845	5	2–14
Women	65–74	73	70,995	10,282	8164–12,941	7	69,138	10	4–21
Women	75–84	108	41,956	25,741	21,306–31,088	12	41,258	29	16–51
Women	85+	125	22,774	54,887	46,054–65,388	7	22,708	31	14–65
Men	≥18	486	503,243	97	88–106	50	497,452	10	8–13
Men	18–34	3	145,893	206	39–635	0	145,102	0	0–3
Men	35–44	11	86,006	1279	683–2321	2	85,001	2	0–9
Men	45–54	33	84,971	3884	2747–5472	1	83,824	1	0–7
Men	55–64	89	72,781	12,229	9926–15,058	9	73,083	12	6–24
Men	65–74	154	68,254	22,563	19,262–26,424	18	66,377	27	17–43
Men	75–84	121	33,709	35,896	30,027–42,895	8	32,641	25	11–49
Men	85+	75	11,629	64,494	51,396–80,852	12	11,424	105	58–186

Both point prevalence and cumulative incidence increased with age, being most common among individuals ≥ 85 years.

The prevalence and cumulative incidence of patients diagnosed with unspecified non-gout crystal arthropathy were higher than that for CPPD with a prevalence of 0.16% (95% CI 0.16–0.17) and an incidence of 15.6 (95% CI 13.4–18.3)/100,000 persons. Compared to women, men had generally both higher prevalence and higher incidence (Table 3).

**Table 3:** Point prevalence and cumulative incidence of other unspecified non-gout crystal arthropathy in Skåne in 2014, age and sex stratified

		Prevalence				Cumulative incidence			
Sex	Age group	Number of persons with diagnosis	Population (*n*)	Prevalence/100,000 persons	95% CI	Number of persons with diagnosis	Population (*n*)	Cumulative Incidence per 100,000 persons	95% CI
All	≥ 18	1662	1,022,545	163	155–171	158	1,011,458	16	13–18
Women	All ≥ 18	734	519,302	141	131–152	71	514,434	8	6–10
Women	18–34	16	143,725	11	7–18	2	143,152	1	0–6
Women	35–44	18	83,016	22	13–35	1	82,328	1	0–8
Women	45–54	45	83,240	54	40–72	3	82,294	4	0–11
Women	55–64	107	73,596	145	120–175	12	73,788	16	9–29
Women	65–74	150	70,995	211	180–250	16	69,053	23	14–38
Women	75–84	215	41,956	512	448–586	24	41,168	58	39–87
Women	85+	183	22,774	804	695–930	13	22,651	57	32–99
Men	All ≥ 18	928	503,243	184	173–197	87	497,024	18	14–22
Men	18–34	19	145,893	13	8–20	5	145,089	4	1–8
Men	35–44	35	86,006	41	29–57	4	84,974	5	1–13
Men	45–54	102	84,971	120	99–146	10	83,762	12	6–22
Men	55–64	165	72,781	227	195–246	14	73,008	19	11–33
Men	65–74	271	68,254	397	352–447	15	66,256	23	13–38
Men	75–84	212	33,709	629	550–719	21	32,549	65	42–99
Men	85+	124	11,629	1067	894–1270	18	11,386	158	89–252

Among the prevalent cases with CPPD and unspecified non-gout crystal arthropathy, 20% and 35% had a joint site-specific diagnosis, respectively. For both CPPD and unspecified non-gout crystal arthropathy, the knee joint was the most commonly affected (74% and 52%, respectively). For CPPD, the second common joint site involved was wrist/hand joint involvement (13%), whereas in patients with unspecified non-gout crystal arthropathy, involvement of the ankles/foot (24%) and wrist/hand (15%) were the second and third most common sites.

Prevalence of CPPD was similar across the educational groups. For unspecified non-gout arthropathies, on the other hand, prevalence decreased with increase of education lever from 0.17% to 0.13%. (Table 4).

**Table 4:** Age- and sex-standardized prevalence of calcium pyrophosphate deposition crystal arthropathy (CPPD) and other non-gout crystal arthropathies and in relation to occupation and educational and income levels

	CPPD	Other non-gout crystal arthropathies
Education	Prevalence (95% CI)	Prevalence (95% CI)
0–9 years	0.09 (0.08–0.10)	0.19 (0.17–0.21)
10–12 years	0.09 (0.08–0.10)	0.17 (0.15–0.18)
13–14 years	0.09 (0.07–0.11)	0.14 (0.11–0.16)
15+ years	0.07 (0.05–0.09)	0.13 (0.11–0.15)
Income		
Lowest	0.08 (0.07–0.10)	0.14 (0.12–0.16)
Middle	0.09 (0.08–0.10)	0.17 (0.16–0.19)
Highest	0.07(0.05–0.08)	0.14 (0.12–0.17)
Occupation		
White, high	0.07 (0.06–0.09)	0.14 (0.12–0.15)
White, low	0.07 (0.06–0.09)	0.16 (0.14–0.18)
Blue, high	0.14 (0.10–0.17)	0.19 (0.15–0.23)
Blue, low	0.09 (0.07–0.11)	0.17 (0.15–0.19)

There were clear patterns of dependence between other socioeconomic variables and prevalence of unspecified non-gout arthropathies or CPPD.

## Discussion

The prevalence of diagnosed non-gout crystal arthropathy is relatively low in the Skåne region in southern Sweden only 0.23% of adults. In agreement with others, we found non-gout crystal arthropathies being almost never diagnosed in younger adults and that both prevalence and incidence were higher in men and increased dramatically with age [1–4]. These findings are in contrast with the results of the ProVA study from Italy by Musacchino et al. reporting that subjects with CC were more frequently women [16]. Some other studies reported CC being more common among women and a female gender as a risk factor [3, 20]. However, other studies investigating the association between gender and non-gout arthropathies (mostly CC) have shown conflicting results. Neame et al. found no association between sex and CC of the knee [11].

Our results are consistent with data from the Swedish study performed in the 1980s where CC was most prevalent in knees and wrists, radiologically [10]. The high occurrence of the location to the knee is also supported by a radiographic examination of subjects attending primary care with knee pain in the UK, in which 7% had radiographic signs of CC. The prevalence of non-gout arthropathies in Skåne was slightly lower than that for symptomatic CC of 0.42% among patients listed at general practitioners [21]. The prevalence of macroscopically visible CPPD crystals in a study on cadaveric knees was 13% [22]. Studies that define CPPD radiographically or macroscopically thus show substantially higher occurrence compared to studies based on clinical signs of CPPD [2]. Recently, a national veteran cohort study from the USA reported a prevalence of CPPD to be 0.52% in patients registered in Veterans health care system, figures that are only slightly higher than our findings likely due to older age in that cohort [23].

Prevalence estimates of CPPD are further complicated as it is closely related to in particular OA and also to some extent to gout [2, 24], both common diseases in the general population.

In the present study, the prevalence of CPPD was similar in all levels of education. In contrast, individuals with < 13 years of education had a lower prevalence of other unspecified non-gout arthropathies than persons with ≥ 15 years of education. Neither the prevalence of unspecified non-gout arthropathies nor CPPD was associated with the level of income. The association with the level of education is similar to that described by us for gout in the same geographical region [25]. The association between educational level unspecified non-gout arthropathies with overall lack of associations with income or occupational exposure may suggest that another lifestyle exposure than occupation may be causally important.

Unspecified non-gout crystal arthropathies and CPPD are diagnosed less often than gout, for which we in the same region have reported a prevalence of ∼ 1.7% [25]. The dramatically higher occurrence with age is on the other hand similar to that shown for gout with the highest prevalence in subjects ≥ 85 years of age [25].

Our study has some limitations. First, misclassifications of diagnoses set by the physicians cannot be excluded since the analyses were performed using diagnostic codes, and we were not able to validate the diagnoses. However, in sensitivity analyses using a more stringent definition, i.e., at least two diagnoses from any physician or at least one from a specialist in rheumatology [26], 86% of all individuals with non-gout crystal arthropathy diagnosis were still identified as cases, rendering some support for validity.

Second, data on occupation status were missing in 22.6% of cases: persons aged 35+ (15.5%), self-employed individuals, or long-term unemployed persons [17]. Since non-gout crystal arthropathies are most common among the elderly people, missing data have probably not influenced the results significantly. Third, missing data of diagnoses in primary care may lead to an underestimation of prevalence and incidence, but since the large majority had at least one visit to a non-primary care physician, this may have had a limited impact on our results.

There are also several strengths of the study. First, the study is a result of a population-based health care register covering an entire regional population, which minimizes the risk for selection bias. In addition, the register data covers a long period from 1998 to 2014. Second, there are only a few previous published epidemiological studies for this group of diseases.

Although our study likely does not take into account asymptomatic radiographic occurrence, it provides robust estimates of diagnosed prevalence and incidence for these groups of diseases and estimates which are scarce in the literature.

## Conclusion

Although considerably less prevalent than gout in southern Sweden, all non-gout crystal arthropathies were diagnosed in 0.23% of the adult population in the Skåne region with an incidence of 21.5 persons per 100,000. Of these, CPPD and other unspecified non-gout crystal arthropathies were the most commonly given diagnoses with a prevalence of 0.09% and 0.16%, respectively. Both prevalence and incidence of these conditions were higher in men and increased with age in both sexes. Non-gout crystal arthropathies also were inversely associated with the level of education.

## Data Availability

The datasets used and analyzed during the current study are available from the corresponding author on reasonable request.

## Abbreviations

SHR: The Skåne Healthcare Register
ICD-10: The International Statistical Classification of Diseases Tenth Revision
CPPD: Calcium pyrophosphate crystal deposition related arthropathy
